# Differences in health-related quality of life among patients after ankle injury

**DOI:** 10.3389/fspor.2022.909921

**Published:** 2022-08-03

**Authors:** Phillip A. Gribble, Rachel E. Kleis, Janet E. Simon, Luzita I. Vela, Abbey C. Thomas

**Affiliations:** ^1^Department of Athletic Training and Clinical Nutrition, College of Health Sciences, University of Kentucky, Lexington, KY, United States; ^2^Division of Athletic Training, Ohio University, Athens, OH, United States; ^3^Department of Health, Human Performance and Recreation, University of Arkansas, Fayetteville, AR, United States; ^4^Department of Kinesiology and Center for Biomedical Engineering and Science, University of North Carolina at Charlotte, Charlotte, NC, United States

**Keywords:** physical activity, ankle sprain, population health, BMI, pain

## Abstract

Ankle sprains are the most common injuries sustained in the physically active, often associated with pain and functional limitations long after initial recovery. In recent years, the impact of ankle sprains on general health and health-related quality of life (HRQoL) has been noted in athletes, but is not well-documented in the general population. We examined differences in HRQoL and general health between individuals with ankle sprain history and healthy controls. Those with ankle sprain reported significantly higher body mass index and general body pain, and lower SF-8 physical component scores than healthy controls. Additionally, there is some indication that physical activity is lower in those with ankle sprain history. This is an important step in illustrating the adverse sequelae of ankle sprains on population health and HRQoL.

## Introduction

Ankle sprains are the most common injury seen in U.S. emergency departments, with over a million visits occurring annually (Lambers et al., 2012; Shah et al., 2016). While ankle sprains are very common in the physically active population (Doherty et al., 2014; Mauntel et al., 2017; Roos et al., 2017; Wiersma et al., 2018; Kerr et al., 2022), approximately 25% of adults in the general population have a history of ankle sprain (Hiller et al., 2012; Baldwin et al., 2017). Unfortunately, in the general population, care for ankle sprains is often limited to symptom management (Bowers et al., 2021; Kosik et al., 2021), and consequently long-term symptoms and physical dysfunction are common following an ankle sprain. Subsequently, those that have sustained a lateral ankle sprain are highly susceptible to developing chronic ankle instability (CAI), a condition characterized by repeated ankle sprains and lingering functional and mechanical ankle instability (Gribble et al., 2013; Miklovic et al., 2018; Delahunt and Remus, 2019; Hertel and Corbett, 2019).

Beyond the detrimental effects at the ankle, the health care burden of ankle sprain history has been discussed more intentionally in recent years, illustrating connections with increased healthcare costs and onset of osteoarthritis (Gribble et al., 2016a,b). Declines in other markers of general health in those with ankle sprain history have been suggested, but most of this work has been performed in young physically active populations (Arnold et al., 2011; Hershkovich et al., 2015; Houston et al., 2015; Hubbard-Turner and Turner, 2015; Holland et al., 2019; Owoeye et al., 2022). Commonly investigated indicators of health include health-related quality of life (HRQoL), pain, physical activity level and body mass index (BMI).

Health related quality of life assesses patient's priorities, goals, values, and perceived wellbeing, while also encompassing their physical, emotional, psychological, and social status (Filbay et al., 2014). It has been established that those with ankle sprain history present with lower levels of self-reported HRQoL (Arnold et al., 2011; Houston et al., 2015; Al Mahrouqi et al., 2020; Kosik et al., 2020). However, much of this work has been focused on ankle region-specific outcomes and instruments, and has been assessed in young adult populations that are more physically active. Because ankle instability is prevalent in the general population, more global investigation of HRQoL in a cross-section of the general population is warranted to understand the broader consequences of ankle injury history.

Pain is a common consequences of ankle sprain history. In a systematic review, Al Adal et al. (2019) report that 50–79% of individuals with CAI have ankle pain up to 6 years post-injury. Arnold et al. (2011) found that individuals with ankle sprain history had worse bodily pain assessed with the SF-36 compared to uninjured controls. Further, >60% of those with ankle sprain history report pain during daily activities or moderate physical activity (Al Adal et al., 2020). While lingering ankle pain could be an expected consequence of previous ankle history, it is unknown to what extent general body pain is present in the general population with ankle sprain history and how that pain may impact HRQoL and other markers of overall health.

Physical activity is an important tenet of overall health, and previous injury history can create challenges to maintaining this aspect of health. Hubbard-Turner and Turner (2015) found lower step counts in young adults with CAI, indicating that ankle sprain history associates with reduced physical activity. However, others have reported that ankle sprain history does not equate with a decline in physical activity (Holland et al., 2019; Al Mahrouqi et al., 2020). The available literature on these connections of ankle injury and physical activity have been mostly focused on young adults and athletes, which makes it difficult to conclude if physical activity is actually disrupted in members of the general population that have sustained an ankle sprain.

Finally, a common surrogate of general health is BMI. As an estimate of body composition, higher BMI suggests high body fat presence (Centers for Disease Control and Prevention^1^), and associates with poor physical function (Han, 1998; Schoffman et al., 2013; O'Neill et al., 2016) and HRQoL (Schoffman et al., 2013), and increased risk of cardiovascular disease (Huxley et al., 2010). Several authors have reported higher BMI in young physically active cohorts with ankle sprain history (Hershkovich et al., 2015; Vuurberg et al., 2019; Svorai Band et al., 2021); but there is some evidence to the contrary (Owoeye et al., 2022). The relationship of ankle sprain history and higher BMI is inconsistent when considering middle-aged cohorts in the general population (Al Mahrouqi et al., 2020; Kosik et al., 2020). Therefore, further investigation is needed to ascertain if BMI is negatively impacted with ankle sprain history.

The primary purpose of this study was to examine markers of general health (pain, physical activity, and BMI) and current HRQoL in a cross-section of the general population with and without a history of self-reported ankle sprains. This will help contribute to the understanding of the potential relationships and negative consequences that musculoskeletal injuries such as ankle sprains have on health in the general population.

## Materials and methods

### Participants

Using a cross-sectional study design and as part of a larger study, an online survey regarding ankle injury history, quality of life, and health outcomes was administered to a random sample of 20,000 adults, ages 18–80, living in the United States. The survey was administered using the ResearchMatch, a national health registry in which volunteers consent to be contacted regarding health studies for which they may be eligible. This registry was created by several academic institutions and supported by the U.S. National Institutes of Health as part of the Clinical Translational Science Award (CTSA) program. Individuals interested in the study were emailed a link to an online survey (Research Suite, Qualtrics, LLC, Provo, UT). Because these data were extracted from a larger study focusing on both knee and ankle injuries, to remove the confounding effect that previous knee injuries may have on HRQoL, anyone who reported a history of knee injury was excluded from the current study. Additionally, this analysis focused on ankle sprains. Therefore, respondents reporting ankle or foot injuries other than an ankle sprain were excluded. The Institutional Review Board at the University of XXXX approved this study.

### Instrumentation

The survey was created from multiple, previously validated questionnaires (Tegner and Lysholm, 1985; Ware et al., 2001; Casanova et al., 2021) and inquired about demographic information, previous injury history and several markers of general health and quality of life. After providing their age, height and mass, participants were asked (yes/no) whether they had previously sustained a lower extremity injury to the ankle, knee, or both joints. Injury was defined as a limitation of desired activities and/or job responsibilities for at least 1 day. If a participant reported that they had a history of a joint injury, they were further asked (yes/no) if the injury(ies) in question required surgical intervention. Additionally, participants indicated (yes/no) if the initial injury evaluation was performed by a medical professional (e.g., physician, physician's assistant, certified athletic trainer, etc.) This analysis focused on respondents that either reported ankle sprains or were free from injury. Those in the Ankle Sprain group answered “yes” to the question if they had sprained their ankle, defined as the ankle “rolling or twisting, resulting in pain, and/or swelling that prevented you from doing what you wanted to do for at least a day”. Those in the Control group answered “no” to having any history of joint injury.

To document general health indicators, participants rated their current physical activity level using the Tegner scale (0–10, where 0 represents complete disability and 10 represents elite-level competitive athletics) (Tegner and Lysholm, 1985). Therefore, higher scores on the Tegner would represent a higher level of perceived physical activity. Additionally, BMI was calculated from self-reported height and mass (Nieto-Garcia et al., 1990; Hattori and Sturm, 2013). The SF-8, a norm-based, 8-item, generic measure that includes scale scores for eight health dimension and two summary scales, the physical and mental component scores (PCS and MCS, respectively) was used to assess HRQoL. Higher SF-8 scores indicate better HRQoL, and reliability (test–retest and other forms) and validity (general healthy populations and clinical populations) analyses of the SF-8 have yielded favorable and psychometrically acceptable results (Ware et al., 2001; Casanova et al., 2021). Versions of the Short Form assessment tools have been used in previous orthopedic research investigating HRQoL in patients with lower extremity musculoskeletal conditions (Bost et al., 2007; Vincent et al., 2010; Arnold et al., 2011; Wright et al., 2017; Gupta et al., 2019). To estimate overall body pain, we selected a single item from the SF-8 that asks respondents to rate their overall body pain in the last week (none [1] to very severe [6]).

### Statistical analysis

Data were exported from Qualtrics into Microsoft Excel (Microsoft Corporation, Redmond, WA) for analysis. Respondents with a history of knee injury and ankle injuries that were not an ankle sprain were excluded, leaving respondents with a history of ankle sprains (Ankle Sprain) or no history of ankle or knee injury (Control).

Shapiro–Wilkes testing indicated that none of the dependent variables were normally distributed (*p* < 0.001). Therefore, Mann–Whitney *U*-tests were employed to compare demographics and survey outcomes between the Ankle Sprain and Control groups. Before comparing the outcomes, Age was considered as a potential co-variate. However, the Ankle Sprain and Control groups were not statistically different (*p* = 0.50) and therefore Age was not used a co-variate in our analyses. The significance level was set a priori at *p* < 0.05. All analyses were conducted using SPSS software (v. 28; IBM Corporation, Armonk, NY).

Additionally, to examine the magnitude of differences between groups, the minimal detectable difference (MDD) values were calculated for each outcome using the equation: MDD = SE^*^√2 × 1.96 (Riemann and Lininger, 2018). Subsequent comparisons were made to determine if the MDD was exceeded between groups in order to describe the potential clinical meaningfulness of any observed differences.

## Results

A total of 3,660 of adults responded to our survey from the random sample of 20,000 (18.3% response rate) that received the survey invitation. Of those, 1,831 respondents reported a history of knee injury and were excluded. Of the remaining 1,829, 277 reported a history of ankle or foot injury that was not an ankle sprain and were excluded. Subsequently, 1,552 respondents were included that either reported a history of an ankle sprain only (Ankle Sprain; *n* = 553) or reported no lower body injury (Control; *n* = 999).

Demographic and survey outcome data between groups are found in Table 1. Height and mass were statistically different between groups, with Ankle Sprain respondents exhibiting greater height and mass than Controls. Ankle Sprain respondents reported statistically higher BMI, Overall Pain, and worse SF-8 PCS scores than Controls (*p* < 0.001 for all outcomes). There was no statistical difference between the groups in reported physical activity (*P* = 0.27) or SF-8 MCS scores (*P* = 0.60). However, when examining the clinical meaningfulness of the group comparisons, the group differences in all outcomes exceeded the MDDs (Table 2), suggesting that Ankle Sprain respondents have higher BMI and overall pain, lower physical activity, and worse SF-8 PCS and MCS scores than non-injured Controls.

**Table 1:** Respondent demographics and group comparisons: Ankle sprain vs. control group.

**Demographic**	**Control** **(*N* = 999)**	**Ankle sprain (*N* = 553)**	**Mann–Whitney *U P*-value**
Sex (males; females)	230; 769	143; 410	N/A
Age (years)	43.19 (15.95)	43.56 (14.75)	0.50
Height (m)	1.67 (0.09)	1.69 (0.10)	0.008
Mass (kg)	75.70 (20.04)	80.65 (22.86)	<0.001
Body mass index (kg/m^2^)	26.90 (6.57)	28.29 (7.52)*	<0.001
Pain	2.47 (1.19)	2.70 (1.21)*	<0.001
Physical activity	4.03 (2.16)	4.22 (2.34)	0.268
SF-8: Physical component score	49.78 (9.44)	48.35 (9.63)*	<0.001
SF-8: Mental component score	46.28 (10.57)	45.27 (10.57)	0.60

*Values represent means (SD). BMI, body mass index; PA, physical activity; PCS, physical component score; MCS, mental component score. Significant difference between groups at P < 0.001*.

**Table 2:** Minimal detectable differences (MDD) for outcome measures between groups.

**Measure**	**MDD**	**LAS vs. control**
Body mass index (kg/m^2^)	0.41	1.39
Pain	0.07	0.23
Physical activity	0.13	0.19
SF-8: Physical component score	0.59	1.43
SF-8: Mental component score	0.66	1.01

*Shaded cells indicate the MDD was exceeded*.

## Discussion

This study examined how individuals from a cross-section of the population with a history of ankle sprains perceive aspects of their general health relative to those with no ankle injury history. Often, ankle sprains are not viewed as a debilitating injury and most individuals return to work and some level of physical activity. However, recent work has illustrated that ankle sprains do associate with lingering ankle instability and a health care financial burden (Gribble et al., 2016a,b); but there is a need to further explore other indicators of possible disruptions in general health that associate with ankle sprain. In this investigation, we focused on a selected group of common indicators of general health (Overall Pain, BMI, Physical Activity and HRQoL) that would provide insight beyond disruptions isolated to the ankle. A unique aspect of this study is that we chose to examine these relationships in a cross-section of the general population, rather than isolated to the more commonly studied young physically active population.

Our analysis supported that those in the general population with ankle sprain history report significantly higher BMI and overall body pain, and lower HRQoL in the physical domain. Our selected clinical significance analysis supported these relationships, as well as lower physical activity and HRQoL in the mental domain in those with ankle sprain history. To our knowledge, this is first study to consider these outcomes in this population, but it is important to compare these data to what has emerged from previous related investigations.

A common, general surrogate of physical health is BMI. Several authors have demonstrated higher BMI in young and middle-aged adults with ankle sprain history (Hershkovich et al., 2015; Vuurberg et al., 2019; Al Mahrouqi et al., 2020; Svorai Band et al., 2021). This is consistent with our statistically significant findings that BMI in the Ankle Sprain group was >1 point higher than the Controls, which was supported by the group difference exceeding the MDD. However, this is in contrast to some work that has reported no influence of ankle sprain history on BMI in young adults (Owoeye et al., 2022) or middle-age adults (Kosik et al., 2020). To our knowledge, our project is the largest to sample BMI using a cross-section of the general population with and without ankle sprain history. Al Mahrouqi et al. (2020) performed a population-based survey with approximately 400 participants with and without chronic ankle pain and an average age of 49 years. They found a larger discrepancy in BMI (>6) between groups than in our data, and their symptomatic chronic ankle pain respondents had an average BMI that was 2 points higher than our respondents with an ankle sprain history. It is possible that pain may play a factor in BMI in those with an ankle sprain history, but more investigation is needed. In contrast, two studies with a much smaller sample size than our current study reported no difference in BMI in young adults (Owoeye et al., 2022) and middle-aged adults (Kosik et al., 2020) with and without ankle sprain history. However, it appears that our results corroborate the majority of the existing literature that supports BMI is higher in those with ankle sprain history (Hershkovich et al., 2015; Vuurberg et al., 2019; Al Mahrouqi et al., 2020; Svorai Band et al., 2021).

Several authors have documented lower levels of HRQoL in those with ankle sprain history using a spectrum of outcome measures (Arnold et al., 2011; Houston et al., 2015; Al Mahrouqi et al., 2020; Kosik et al., 2020). The majority of these measures are ankle-region specific, which may not capture aspects of the individuals' global HRQoL. We chose to employ global measures with the SF-8 PCS and MCS. The ankle sprain history respondents had significantly worse physical component HRQoL scores than controls, and the group differences in both physical and mental component scores exceeded the MDDs. These differences in global HRQoL observed in those with ankle sprain history in the general population is consistent with other investigations. Arnold et al. (2011) reported worse SF-36 scores in young adults with ankle sprain history, and Al Mahrouqi et al. (2020) observed worse scores on the Assessment of Quality of Life instrument in a cross-section of the general population. While there are numerous methods to illustrate this facet of general health, it appears that our selected approach contributes to the literature that those with ankle sprain history are likely to develop a diminished level of HRQoL.

It is important to note that not only did the Ankle Sprain respondents report worse SF-8 PCS scores than the Control respondents, but their physical wellbeing scores were also lower than established normative data in healthy adults (approximate PCS score of 50; Lefante et al., 2005). The observed physical function limitations in members of the general population with ankle sprain history highlight the importance of addressing the HRQoL deficiencies in ankle sprain patients which may be lacking with the current standard of care. It is important to note that additional factors beyond the scope of this study could impact these observed results including pre-injury levels of HRQoL, total number of ankle sprains and time since the most recent injury, and other concomitant injury other than knee injuries. We look forward to continued investigation in this aspect of ankle sprain consequences.

Those with acute ankle sprains will have a chief complaint of debilitating pain that limits function, which often subsides in the short-term (Gribble, 2019). Unfortunately, many with an ankle sprain history will experience lingering, chronic ankle pain (Al Adal et al., 2019). Ankle specific pain is an important factor that can link to loss of ankle function, but there is value in considering overall body pain in those with ankle sprain history as a contributor to potential general health decline. The respondents in our study with ankle sprain history from the general population reported significantly higher levels of overall body pain, and this group difference exceeded the MDD. This relationship is consistent with other reports of worse body pain the general population with ankle sprain history (Arnold et al., 2011) as well as a report that the majority of individuals with ankle sprain history report pain during daily activities or moderate physical activity (Al Adal et al., 2020). Given the alarming trends in pharmacological approaches to addressing pain in ankle sprain patients (Bowers et al., 2021; Kosik et al., 2021), both ankle specific and overall body pain needs to be addressed further in this facet of the population.

Previous reports have linked pain and a reduction in physical activity (Landmark et al., 2011, 2013). Although the Ankle Sprain respondents reported a lower level of physical activity and this group difference exceeded the MDD, it was not a statistically significant difference. Therefore, we feel there is a need for caution in concluding a decline in physical activity levels in the general population with an ankle sprain history. The literature is inconsistent in supporting a negative influence of ankle sprain history on physical activity. Hubbard-Turner and Turner (2015) used step counts to illustrate a reduced level of physical activity in young adults with ankle sprain history. In contrast, two studies that have used the International Physical Activity Questionnaire (IPAQ) have not substantiated a reduced level of physical activity in young adults (Holland et al., 2019) and middle-age adults (Al Mahrouqi et al., 2020) with ankle sprain history. We utilized the Tegner Activity Scale, which has been used more commonly in knee-injured populations (Tegner and Lysholm, 1985; Briggs et al., 2009a,b), but to our knowledge has not been applied in an ankle sprain history population. The selection of the Tegner was, in part, due to the design of the larger study that did include analysis of knee-injured patients (Bruce et al., 2017; Thomas et al., 2019; Kleis et al., 2020). There may be some limitations in applying the Tegner to an ankle sprain history population, and based on other published literature mentioned above, future investigations may need to employ other instruments to quantify physical activity in this injury population of the general population across the lifespan.

A lack of understanding of the interplay between ankle sprain history on overall health and HRQoL presents challenges to implement comprehensive and effective management programs (Parsons and Snyder, 2011). It is well-known that higher BMI increases the risk of developing posttraumatic osteoarthritis, obesity and its related comorbidities, all of which are associated with decreased HRQoL, and pain is a common co-existing factor. We did not document osteoarthritis in this analysis, but our findings of higher BMI and overall body pain along with decreased HRQoL should stimulate more investigation into the comprehensive general health consequences of ankle sprain and the need for more successful mitigation strategies. This will underscore the importance of the health care provider in treatment and education of the deleterious effects of ankle sprain history to contribute to better outcomes for the ankle sprain patient (Centers for Disease Control Prevention, 2011).

This study is not without limitations. First, because this was an online based survey, the study was biased to respondents that had access to internet and a reliable mobile device or computer. Next, the design of our study did not involve access to complete medical records from the respondents, instead relying on subjective participant reporting, which is subject to recall bias. Inherent to this study design, factors such as time of injury, number of ankle injuries, the precise injury sustained, pre-injury health status, time since injury, and other injuries besides the excluded knee injuries could not be extracted and included in our analysis. However, even with the absence of complete medical records, the respondents' data provide valuable outcomes that suggest markers of overall health are likely diminished in the general population with an ankle sprain history. Another possible limitation in our study was the utilization of the Tegner instrument to rate physical activity. This questionnaire was selected because it assesses physical activity on a broad scale; but it is possible that may not have been the optimal outcome tool for this analysis. Additional investigation may be needed to fully illustrate physical activity levels in those with and without ankle sprain history in the general population. Finally, our study does have some potential sources of bias, and the cross-sectional study design of our study limits any conclusions of cause and effect of ankle sprain on diminished health outcomes. In spite of this, the data from this project provide important initial illustrations of selected key associations that may be present in the general population with ankle sprain history. Our study emphasizes the need for ankle injury prevention, treatment, and patient education strategies that successfully reduce negative health outcomes in those that have sustained this common musculoskeletal injury. We look forward to future studies that will explore successful strategies to address these likely negative connections between ankle sprain and diminished population health.

## Conclusion

Those in the general population with a history of ankle sprain present with clinically meaningful differences in BMI, pain, physical activity and HRQoL compared with those that have never experienced a lower extremity musculoskeletal injury. This suggests there may be an association between ankle sprain history and reduced markers of general health. Our findings will help improve the understanding of the impact of musculoskeletal injuries on long-term health burdens, highlighting the need for improved prevention, management, and rehabilitation strategies particularly for ankle sprains.

## Data availability statement

The original contributions presented in the study are included in the article/supplementary materials, further inquiries can be directed to the corresponding author/s.

## Ethics statement

The studies involving human participants were reviewed and approved by University of Kentucky. The patients/participants provided their written informed consent to participate in this study.

## Author contributions

PG, RK, JS, LV, and AT contributed to the conception, analysis, and writing of this manuscript. All authors contributed to the article and approved the submitted version.

## Conflict of interest

The authors declare that the research was conducted in the absence of any commercial or financial relationships that could be construed as a potential conflict of interest.

## Publisher's note

All claims expressed in this article are solely those of the authors and do not necessarily represent those of their affiliated organizations, or those of the publisher, the editors and the reviewers. Any product that may be evaluated in this article, or claim that may be made by its manufacturer, is not guaranteed or endorsed by the publisher.

## Footnotes

^1^*About Adult BMI*. Available online at: https://www.cdc.gov/healthyweight/assessing/bmi/adult_bmi/index.html.
